# What is the potential impact of genetic divergence of plastid ribosomal genes between *Silene nutans* lineages in hybrids? An *in silico* approach using the 3D structure of the plastid ribosome

**DOI:** 10.3389/fpls.2023.1167478

**Published:** 2023-05-08

**Authors:** Zoé Postel, Théo Mauri, Marc F. Lensink, Pascal Touzet

**Affiliations:** ^1^ Univ. Lille, CNRS, UMR 8198 - Evo-Eco-Paleo, Lille, France; ^2^ Univ. Lille, CNRS, UMR 8576 – UGSF - Unité de Glycobiologie Structurale et Fonctionnelle, Lille, France

**Keywords:** plastid ribosome, protein structure, protein-protein interaction, ribosomal gene evolution, plastid-nuclear incompatibilities

## Abstract

**Introduction:**

Following the integration of cyanobacteria into the eukaryotic cells, many genes were transferred from the plastid to the nucleus. As a result, plastid complexes are encoded both by plastid and nuclear genes. Tight co-adaptation is required between these genes as plastid and nuclear genomes differ in several characteristics, such as mutation rate and inheritance patterns. Among these are complexes from the plastid ribosome, composed of two main subunits: a large and a small one, both composed of nuclear and plastid gene products. This complex has been identified as a potential candidate for sheltering plastid–nuclear incompatibilities in a Caryophyllaceae species, Silene nutans. This species is composed of four genetically differentiated lineages, which exhibit hybrid breakdown when interlineage crosses are conducted. As this complex is composed of numerous interacting plastid–nuclear gene pairs, in the present study, the goal was to reduce the number of gene pairs that could induce such incompatibilities.

**Method:**

We used the previously published 3D structure of the spinach ribosome to further elucidate which of the potential gene pairs might disrupt plastid–nuclear interactions within this complex. After modeling the impact of the identified mutations on the 3D structure, we further focused on one strongly mutated plastid–nuclear gene pair: rps11–rps21. We used the centrality measure of the mutated residues to further understand if the modified interactions and associated modified centralities might be correlated with hybrid breakdown.

**Results and discussion:**

This study highlights that lineage-specific mutations in essential plastid and nuclear genes might disrupt plastid–nuclear protein interactions of the plastid ribosome and that reproductive isolation correlates with changes in residue centrality values. Because of this, the plastid ribosome might be involved in hybrid breakdown in this system.

## 1 Introduction

Plastids come from ancient cyanobacteria that integrated eukaryotic cells as endosymbionts roughly a billion years ago (Gray, 1999). After this integration, this organelle transferred a certain amount of its genes to the nucleus, ending up encoding only a few of the original gene set (Sloan et al., 2018). These remaining 120 or so genes are involved in photosynthesis and housekeeping function in the plastid (Zoschke and Bock, 2018). Due to these transfers, the essential plastid protein complexes are encoded both by plastid genes and nuclear genes whose gene products are targeted to the plastid (henceforth called nuPt). Plastid and nuPt genes need to interact with one another for correct protein complex function (Rand et al., 2004; Greiner and Bock, 2013; Zhang et al., 2015). Nuclear and plastid genomes have contrasting features, such as differences in mutation rate, which is much lower in the plastid (Smith, 2015), or different inheritance patterns, with biparental inheritance for the nuclear genome and maternal inheritance for the plastid genome (Greiner et al., 2014). As such, any mutation occurring in one of the two partners will generate strong selective pressure for the fixation of compensatory mutation in the other one (Greiner and Bock, 2013). Tight co-adaptation between interacting plastid and nuPt genes is therefore required and indeed enforced (Forsythe et al., 2021). Independent accumulation of mutations in both plastid and nuPt genes can occur in isolated lineages or populations (Levin, 2003). When and if hybridization occurs between these isolated lineages, co-adaptation between nuPt and plastid genes will be disrupted in hybrids (Sloan et al., 2018). Indeed, hybridization will bring together a plastid genome mismatched with half of the hybrid nuclear background, leading to a potential hybrid breakdown (i.e., decrease in fertility and survival) due to plastid–nuclear incompatibilities (PNIs) (Greiner et al., 2011). These incompatibilities are thought to be part of the first post-zygotic reproductive barriers that emerged, as they can lead to reproductive isolation (RI) between lineages through a decrease in hybrid fitness (Barnard-Kubow et al., 2016). When such incompatibilities are involved in speciation (i.e., the process leading to RI; Matute and Cooper, 2021), RI is asymmetric in reciprocal crosses, depending on the lineage that is the plastid donor (Turelli and Moyle, 2007; Burton and Barreto, 2012). Involvement of PNIs as reproductive barriers have already been studied in some plant systems (*Campanulastrum americanum* (Barnard-Kubow et al., 2016), *Pisum sativum* (Bogdanova et al., 2015), *Silene nutans* (Postel et al., 2022), and *Oenothera* spp. (Zupok et al., 2021)). They arise as byproduct of the independent accumulation of mutations either in plastid or nuPt genes during lineages divergence in allopatry (Greiner and Bock, 2013; Postel and Touzet, 2020). Yet, molecular mechanisms and the identification of co-adapted pairs of genes are still largely missing (but see Zupok et al., 2021).

PNIs were also potentially involved in RI between lineages of *Silene nutans* (Caryophyllaceae) (Postel et al., 2022). This species is composed of several genetically differentiated lineages in France based on plastid sequences and nuclear microsatellite markers, and their geographic distribution in Europe reflects colonization from past glacial refugia (Martin et al., 2016; Van Rossum et al., 2018). Diallele crosses between four of these lineages, an eastern one E1 and three western one (W1, W2, W3) revealed strong and asymmetric RI between them (Martin et al., 2017; Van Rossum et al., in prep). Analysis of plastid genetic diversity and nuPt genes in these four lineages uncovered lineage-specific co-evolution patterns between plastid and nuPt genes that could result in PNIs in hybrids (Postel et al., 2022). Candidate gene pairs for PNIs were identified in the plastid ribosomes (Postel et al., 2022), a plastid complex whose components are a large and a small subunit, encoded both by nuPt and plastid genes (Bieri et al., 2017). Plastid ribosomes are an essential component for plant development and growth (Robles and Quesada, 2022). For example, it is involved in translation of essential plastid genes dealing with photosynthesis and plastid gene expression (Tiller and Bock, 2014), and evidence is accumulating for the involvement of ribosomal protein in many other plant biological aspects (e.g., plastid biogenesis, embryogenesis, etc.) (Robles and Quesada, 2022). Plant mutants for plastid ribosomal proteins exhibit various phenotypes, such as seedling lethality, misshapen leaves, and chlorosis, highlighting the importance of having a functional plastid ribosome for correct plant development and growth. Because of its functional importance, any mutations in plastid ribosomal genes might have drastic consequences on a plant’s fitness.

Plastid and nuPt genes encoding plastid ribosome exhibited the largest amount of lineage-specific nonsynonymous (NS) mutations (i.e., mutations leading to a change of the encoded amino-acid) and elevated dN/dS (i.e., proportion of nonsynonymous (*N*) and synonymous (*S*) mutations on the total number of *N* and *S* sites) (Postel et al., 2022). Elevated dN/dS was thought to be the result of positive selection on the plastid genes and on some nuPt genes (Postel et al., 2022). Regarding the nuPt genes, dN/dS was significantly higher compared to nuclear genes encoding the cytosolic ribosome (i.e., gene products not targete to the plastid), suggesting that this increase in the number of NS mutations might be the result of plastid–nuclear co-evolution (Postel et al., 2022). Some of the NS mutations identified in plastid and nuPt genes encoding the large and small plastid ribosomal subunit were directly located at the protein residue contact position, suggesting structurally mediated co-evolution between plastid and nuPt genes within the plastid ribosome (Havird et al., 2015; Postel et al., 2022).

Many plastid–nuclear gene pairs encoding subunits of the plastid ribosome were thus identified as potential candidates for PNIs between lineages of *S. nutans* (Postel et al., 2022). To further identify which of these pairs could be responsible for PNIs, we used the crystallographic structure of the spinach (*Spinacia oleracea*) plastid ribosome (Sharma et al., 2007) to assess the impact of the NS mutations identified in each plastid and nuPt gene of these pairs on the residue contact interactions between plastid and nuclear proteins within the large and small plastid ribosomal subunits. We modeled the different NS mutations for each lineage and each nuclear and plastid candidate proteins on these subunits to further narrow down the list of PNI candidates in the plastid ribosome. This led us to focus on the most impacted plastid–nuclear gene pair (*rps11*–*rps21*), transforming models into graphs called residue interactions networks (RINs) in order to calculate centralities of the residues (Brysbaert et al., 2018). Considering protein structures, a network of interconnected nodes (residues), the so-called central nodes are those residues with the most influence in the network; residue centralities have previously been shown to highlight residues important for protein structure and function (Del Sol et al., 2006; Hu et al., 2014; Trouvilliez et al., 2022). We then performed principal component analysis on the residue centralities in order to investigate a potential correlation between the modification of residue centralities and the interlineage hybrid breakdown in *Silene nutans*.

## 2 Materials and methods

### 2.1 Identification of mutations

Mutation identification was previously done in Postel et al. (2022). Briefly, we searched for all mutations differently fixed between lineages of *Silene nutans*, in plastid and nuPt gene sequence alignments of the plastid ribosomal proteins, using an in-house biopython script (https://github.com/ZoePos/Variants-dectections) (Postel et al., 2022). We then aligned the plastid and nuPt gene sequences of *S. nutan*s with the one of *Spinacia oleracea*, used as reference. The spinach structure and the associated protein sequences were available in Protein Data Bank Europe (PDBe) (PDB id: 5MMM) and contains 60 chains corresponding to the different ribosomal proteins of the small (rps) and large (rpl) subunits and some RNA strands (Sharma et al., 2007). After aligning *S. nutans* and *S. oleracea* sequences, we compared the encoded amino-acid between *S. nutans* and the spinach at each position containing mutations between lineages of *S. nutans*. We reported the different mutations identified in lineages of *S. nutans* and the corresponding amino acid in *S. oleracea* in **Supplementary Tables 1, 2.**

### 2.2 Identification of impacted interactions between the subunits

To identify interactions between subunits inside the plastid ribosome, the structure was transformed into a RIN. A RIN is a graphical representation of the structure where nodes represent the residues and the edges represent the interactions between residues. To define an interaction, any one atom of residue A must be at a distance between 2.5 and 5 Ångström (Å) from any one atom of residue B. Detected interactions were then exported into a text file with the two amino acids involved and the minimal distance between the two. From this file, only interactions between plastid and nuPt genes within each of the large and small ribosomal subunits were analyzed in order to identify potentially impacting mutations.

### 2.3 Identification of the interaction type and possible modifications

To identify the type of interaction and the potential impact of the mutations on the interaction, mutations were modeled based on the spinach reference structure. For this, the PyMOL software with the mutagenic tool was used (DeLano, 2002; Schrödinger and DeLano, 2020). This tool can replace an amino acid with another one by transforming the lateral chain; it also allows for the selection of the most optimal rotamer. From this, an atomic point of view of the interaction can be deduced and the different types of interaction determined. We define the following four types of interactions: hydrogen bond, hydrophobic, salt bridge, and polar. The mutation can lead to a change in the type of interaction, the creation of a new one, or the loss of the interaction. The type of interaction was determined through visual inspection. We finally chose to focus on one plastid–nuclear gene pair: *rps11* (plastid encoded)–*rps21* (nuclear encoded), which accumulated the highest number of mutations and subsequent interaction modifications.

### 2.4 Creation of the different models

**Figure 1:**
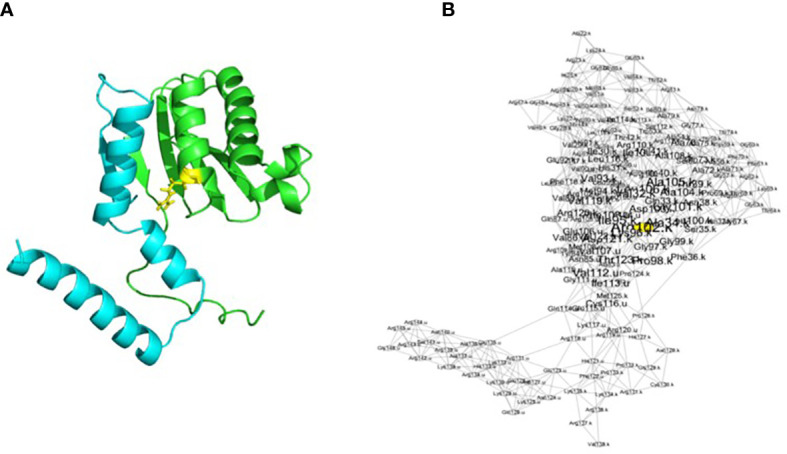
Representation of the *rps11–rps21* proteins as a structure **(A)** and as a RIN **(B)** in *S. oleracea*. The blue chain is *rps21*, and the green chain is *rps11*. The yellow residue corresponds to the yellow node in the networks and corresponds to a residue with a centrality of ≥2. Visualization of the network has been made with Cytoscape after running RINspector (Shannon et al., 2003; Brysbaert et al., 2018).

We created 16 different models considering the different cross types and directions, i.e., E1_E1, E1_W1, E1_W2…W3_W2, and W3_W3, later called plastid–nuclear combinations (e.g., for E1_W1, we modeled the mutations identified in the lineage E1 in the plastid genes and the mutations identified in the lineage W1 in the nuPt genes). These three-dimensional models were based on the spinach ribosome structure complex resolved in 2007 (Sharma et al., 2007). Each one contained the associated mutations on the genes *rps11* and *rps21* described in **Table 1**. The different mutations were created with PyMOL using the mutagenesis tool. Even though the interface permits selecting the optimal side chain rotamer, we performed additional energy minimizations to improve side chain packing. The models were minimized using the YASARA software with YASARA minimization (Land and Humble, 2018) (**Figure 1**).

**Table 1:** Detail of the interactions found between candidate gene pairs, with the impact of the mutation in one of the two partners on the interaction.

Plastid						Nuclear						Dist (Å)	Interaction type	
Gene	Residue	Amino acid of				Gene	Residue	Amino acid of					Before mutation	After mutation
E1		W1	W2	W3	E1			W1	W2	W3	E1			
*rpl32*	Arg49	L	L	P	L	*rpl17*	Tyr122	–^a^	–^a^	–^a^	–^a^	4.37	H bridge	Ø
*rpl14*	Arg104	G	–^a^	–^a^	–^a^	*rpl19*	Val155	–^a^	–^a^	–^a^	–^a^	4.17	Ø	Ø
							Glu157	–^a^	–^a^	–^a^	–^a^	4.85	Ø	Ø
							Ser163	–^a^	–^a^	–^a^	–^a^	2.98	Polar	Ø
							Tyr165	–^a^	–^a^	–^a^	–^a^	2.99	Polar	Ø
*rps3*	Lys146	–^a^	–^a^	–^a^	–^a^	*rps5*	Val198	–^a^	–^a^	–^a^	I	3.86	Ø	Ø
*rps11*	Pro98	–^a^	S	S	–^a^	*rps21*	Ile113	–^a^	–^a^	–^a^	–^a^	3.47	Ø	Ø
	Leu116	–^a^	–^a^	–^a^	V		Cys116	–^a^	–^a^	–^a^	–^a^	3.86	Ø	Ø
		–^a^	–^a^	–^a^	–^a^		Val90	–^a^	–^a^	–^a^	–^a^	3.34	Ø	Hydrophobic
	Ser117	–^a^	–^a^	–^a^	–^a^		Glu94	–^a^	–^a^	–^a^	–^a^	3.76	Ø	Ø
		–^a^	–^a^	–^a^	–^a^		Leu99	–^a^	–^a^	–^a^	–^a^	3.53	Hydrophobic	Hydrophobic
	Phe118	–^a^	–^a^	–^a^	–^a^		Val88	I	–^a^	–^a^	–^a^	4.57	Polar	Polar
		–^a^	–^a^	–^a^	–^a^		Leu89	F	–^a^	–^a^	S	3.41	Ø	Ø
		–^a^	–^a^	–^a^	–^a^		Val88	I	–^a^	–^a^	–^a^	3.73	Ø	Hydrophobic
		–^a^	–^a^	–^a^	–^a^		Leu89	F	–^a^	–^a^	S	3.44	Hydrophobic	Hydrophobic/Ø
	Val119	–^a^	–^a^	–^a^	–^a^		Val88	I	–^a^	–^a^	–^a^	3.03	Polar	Polar
	Pro132	–^a^	–^a^	–^a^	–^a^		Tyr121	H	H	–^a^	H	3.67	Ø	Ø
	Pro133	–^a^	–^a^	–^a^	–^a^							3.49	Ø	Ø
	Lys134	–^a^	–^a^	–^a^	–^a^							3.51	Ø	Polar
	Lys135	–^a^	–^a^	–^a^	–^a^							3.79	Ø	H bridge
	Lys135	–^a^	–^a^	–^a^	–^a^		Glu127	–^a^	D	D	D	3.12	Salt bridge	Salt bridge
	Arg136	–^a^	–^a^	–^a^	–^a^		Tyr121	H	H	–^a^	H	3.3	H bridge	H bridge
*rps19*	Arg65	–^a^	–^a^	Y	D	*rps13*	Arg124	–^a^	–^a^	–^a^	–^a^	2.64	H bridge	H bridge
							Glu127	–^a^	–^a^	–^a^	Q	2.75	Salt bridge	Ø/Ø
							Ile128	–^a^	–^a^	–^a^	–^a^	4.5	H bridge	Ø
*rps18*	Arg50			Q		*rps21*	Arg139	–^a^	–^a^	–^a^	–^a^	3.6	H bridge	Ø
							Asn140	–^a^	–^a^	–^a^	–^a^	4.12	H bridge	H bridge
							Arg143	–^a^	–^a^	–^a^	–^a^	3.36	H bridge	Ø

In purple: loss of the interaction with the mutation. In pink: creation of a new interaction with the mutation. In green: residue of rps11 for which centrality was calculated. In blue: residue of rps21 for which centrality was calculated.

Dist, distance between amino-acid residues in angstrom.

a No mutations compared to the reference sequence of S. oleracea.

### 2.5 Creation of the residue interaction networks from the models and centrality analyses

RINs were created for each model for a total of 16 RINs using ringraph, an in-house C program that calculates distances between amino acids as described above (**Figure 1**). From these networks, it is possible to calculate centralities of nodes thanks to graph theory. A residue with a high centrality represents a residue that connects other residues together within a protein network (here, within proteins RPS11 and RPS21 and their interactions). The more a residue contributes to residue connection within a structure, the more it is central and has an important structural role. Different types of centrality can be calculated by the same in-house program. In order to obtain the highest number of central residues, we decided to calculate four different kinds of accessibility: betweenness, closeness, degree, and eigenvector (**Table 2**) (Brysbaert and Lensink, 2021). We looked at the difference in centrality for the highlighted residues. We then calculated a centrality score (i.e., a Z-score), which is normalized with the size of the network. A residue with a high Z-score (≥2) is considered central. The results of all centralities for the 16 models were retrieved and imported into a CSV file.

**Table 2:** Summary of the types of centrality calculations used and their methods.

Centrality measure	Method
Betweenness centrality analysis (BCA)	BCA highlights residues often found in the minimal path between every residue.
Closeness centrality analysis (CCA)	CCA is calculated as the reciprocal of the sum of the lengths of the shortest paths between the node and all other nodes in the graph
Degree centrality analysis (DCA)	DCA calculates centrality based on the number of nodes connected to the residue analyzed.
Eigenvector centrality analysis (ECA)	ECA calculates the centrality of nodes based on the centrality of other nodes, meaning that a node connected to a high-centrality node will have a higher centrality.

### 2.6 Principal component analysis on centralities

To see if modification of residue centralities associated with the plastid–nuclear combinations could explain the outcomes of interlineage crosses, principal component analysis (PCA) was conducted using the centrality values of *rps11–rps21* residues for each cross type and the five different measures of centrality. Because the results for the five centrality measures were similar, we only reported on the BCA and CCA centrality measures (Amitai et al., 2004; Hu et al., 2014). PCA was implemented in an R-Script (R version 3.6.3) with RStudio and R packages (table.data V1.2.0 for data analysis and factoextra V1.0.7 for representation). PCA was calculated with the “prcomp” command. The contributions of each variable and each individual were calculated and retrieved in CSV files.

## 3 Results

### 3.1 Modification of interactions due to mutation

Numerous lineage-specific NS mutations had been identified in interacting plastid and nuPt genes encoding the plastid ribosomes within *S. nutans* lineages (Postel et al., 2022) (**Supplementary Tables S1, S2**). Mutation selection led to a subset of 28 mutations with their associated modified interaction (**Table 1**). In total, we observed eight losses of interaction, four gains of interaction, and 16 mutations without a change in interaction type (**Table 1**). Among the mutations leading to the loss of interactions, four led to the loss of a hydrogen bridge between residues, two to the loss of a polar interaction, one to the loss of a hydrophobic interaction, and the last one to the loss of a salt bridge (**Table 1**, in purple). Among the interactions that were gained through mutation in one of the two partners, two were new hydrophobic interactions, one polar interaction, and one hydrogen bridge (**Table 1**, in pink).

A large majority of the mutations inducing a change in the interaction between plastid and nuPt genes were located on genes *rps11* (plastid encoded) and *rps21* (nuclear encoded). This gene pair is also the one that contained most of the mutations (i.e., 28 in total) (**Table 1**; **Supplementary Table S2**). We therefore focused our attention on this gene pair in subsequent analyses.

### 3.2 RIN analysis of mutations for the *rps11–rps21* gene pair

We looked at the centralities of the mutated residues in *rps11–rps21* genes for the lineages of *S. nutans* to see if the mutations could impact the stability of the different subunits of the ribosome. In the main text, for the sake of clarity, we only presented the figure results of the BCA and CCA analyses. Additional figures for the three other centrality measures can be found in the **Supplementary Data**.

Focusing first on BCA result for *rps11* residues, when both E1 and W3 are the maternal parents, residue 98 showed a decrease in centrality (**Table 3**). For residue 116, such a decrease is only observed when W3 is the mother (**Table 3**). For the cross-direction E1_E1, W1_E1, W2_E1, W3_W1, and W3_W3, we can observe changes in centrality (BCA) for residue 117 (serine) and residues 118, 119, and 132, but to a lesser extent (**Table 3**). We did not observe any changes in centrality associated with the cross types and directions for the other residues of this gene. Regarding the result for the nuclear gene, *rps21*, no changes in centrality associated with either cross type or direction were observed except for residue 113 (**Table 3**). In this case, similarly to what was observed for residue 117 in *rps11*, loss of centrality was observed for the crosses E1_E1, W1_E1, W2_E1, W3_W1, and W3_W3 (**Table 3**).

**Table 3:** BCA and CCA centrality score of mutated and interacting residues of *rps11* and *rps21* according to the 16 different plastid–nuclear combinations.

Crosses	*rps11*										*rps21*						
	98.*k* ^a^	116.*k* ^a^	117.*k*	118.*k*	119.*k*	132.*k*	133.*k*	134.*k*	135.*k*	136.*k*	88.*u* ^a^	89.*u* ^a^	90.*u*	113.*u*	116.*u*	121.*u* ^a^	127.*u* ^a^
BCA																	
E1 vs. E1	0.52	0.25	−0.14	−0.39	0.38	−0.13	−0.47	−0.57	−0.34	−0.27	1.86	1.57	−0.42	−0.14	−0.69	0.94	−0.53
E1 vs W1	0.51	0.21	0.42	−0.45	0.55	−0.13	−0.47	−0.58	−0.34	−0.27	1.85	1.56	−0.39	0.07	−0.67	0.95	−0.53
E1 vs. W2	0.53	0.21	0.40	−0.46	0.55	−0.14	−0.48	−0.58	−0.35	−0.28	1.91	1.73	−0.40	0.07	−0.68	0.97	−0.50
E1 vs. W3	0.50	0.18	0.41	−0.45	0.53	−0.20	−0.50	−0.58	−0.34	−0.27	1.93	1.53	−0.39	0.07	−0.66	0.95	−0.53
W1 vs. E1	0.75	0.23	−0.16	−0.4	0.39	−0.14	−0.49	−0.59	−0.35	−0.28	1.88	1.86	−0.43	−0.15	−0.71	0.97	−0.51
W1 vs. W1	0.71	0.19	0.47	−0.45	0.54	−0.13	−0.48	−0.58	−0.34	−0.28	1.89	1.65	−0.39	0.09	−0.67	0.95	−0.53
W1 vs. W2	0.70	0.18	0.41	−0.46	0.54	−0.19	−0.50	−0.58	−0.34	−0.27	1.97	1.64	−0.39	0.07	−0.67	0.96	−0.53
W1 vs. W3	0.71	0.18	0.40	−0.45	0.54	−0.13	−0.48	−0.58	−0.34	−0.27	1.98	1.64	−0.39	0.07	−0.67	0.95	−0.53
W2 vs. E1	0.70	0.22	−0.14	−0.39	0.35	−0.20	−0.50	−0.58	−0.34	−0.27	2.10	1.61	−0.43	−0.18	−0.69	0.97	−0.53
W2 vs. W1	0.71	0.19	0.47	−0.45	0.54	−0.13	−0.48	−0.58	−0.34	−0.28	1.89	1.65	−0.39	0.09	−0.67	0.95	−0.53
W2 vs. W2	0.71	0.18	0.41	−0.45	0.53	−0.13	−0.48	−0.58	−0.34	−0.27	1.96	1.64	−0.39	0.07	−0.67	0.95	−0.53
W2 vs. W3	0.71	0.18	0.40	−0.45	0.54	−0.13	−0.48	−0.58	−0.34	−0.27	1.98	1.64	−0.39	0.07	−0.67	0.95	−0.53
W3 vs. E1	0.44	−0.09	0.44	−0.46	0.69	−0.20	−0.51	−0.59	−0.35	−0.28	1.77	1.68	−0.40	0.09	−0.68	0.97	−0.51
W3 vs. W1	0.52	0.05	−0.11	−0.4	0.50	−0.13	−0.48	−0.58	−0.34	−0.28	1.86	1.58	−0.42	−0.11	−0.69	0.94	−0.53
W3 vs. W2	0.53	−0.01	0.40	−0.47	0.67	−0.14	−0.48	−0.59	−0.35	−0.28	1.86	1.75	−0.40	0.08	−0.69	0.97	−0.51
W3 vs. W3	0.59	−0.05	−0.12	−0.39	0.51	−0.13	−0.47	−0.58	−0.34	−0.27	1.86	1.57	−0.42	−0.12	−0.69	0.94	−0.53
CCA																	
E1 vs. E1	1.49	1.00	0.37	0.58	1.29	−0.60	−0.65	−1.23	−1.13	−1.26	1.22	1.02	0.20	0.67	−0.04	−0.26	−1.57
E1 vs. W1	1.48	0.99	0.45	0.57	1.36	−0.60	−0.66	−1.23	−1.13	−1.27	1.22	1.02	0.27	0.76	0.02	−0.27	−1.58
E1 vs. W2	1.49	0.99	0.45	0.57	1.35	−0.60	−0.66	−1.21	−1.11	−1.24	1.21	1.00	0.27	0.75	0.03	−0.25	−1.57
E1 vs. W3	1.48	0.99	0.45	0.57	1.36	−0.61	−0.67	−1.23	−1.13	−1.27	1.22	0.99	0.27	0.77	0.04	−0.27	−1.59
W1 vs. E1	1.51	0.97	0.35	0.55	1.28	−0.58	−0.64	−1.22	−1.11	−1.25	1.22	1.04	0.18	0.66	−0.05	−0.25	−1.56
W1 vs. W1	1.49	0.99	0.46	0.58	1.35	−0.59	−0.65	−1.22	−1.12	−1.26	1.23	1.03	0.28	0.75	0.03	−0.26	−1.57
W1 vs. W2	1.49	0.99	0.45	0.57	1.36	−0.61	−0.67	−1.23	−1.13	−1.27	1.23	1.01	0.27	0.76	0.03	−0.27	−1.59
W1 vs. W3	1.49	1.00	0.45	0.57	1.36	−0.61	−0.67	−1.22	−1.12	−1.26	1.23	1.00	0.28	0.76	0.03	−0.26	−1.58
W2 vs. E1	1.48	1.00	0.38	0.59	1.32	−0.61	−0.67	−1.22	−1.12	−1.25	1.27	1.01	0.29	0.66	−0.03	−0.25	−1.58
W2 vs. W1	1.49	0.99	0.46	0.58	1.35	−0.59	−0.65	−1.22	−1.12	−1.26	1.23	1.03	0.28	0.75	0.03	−0.26	−1.57
W2 vs. W2	1.49	1.00	0.45	0.57	1.36	−0.61	−0.67	−1.23	−1.12	−1.26	1.23	1.01	0.27	0.76	0.03	−0.26	−1.58
W2 vs. W3	1.49	1.00	0.45	0.57	1.36	−0.61	−0.67	−1.22	−1.12	−1.26	1.23	1.00	0.28	0.76	0.03	−0.26	−1.58
W3 vs. E1	1.46	0.79	0.46	0.58	1.37	−0.59	−0.65	−1.22	−1.12	−1.26	1.14	0.96	0.28	0.77	0.04	−0.26	−1.57
W3 vs. W1	1.48	0.81	0.39	0.59	1.29	−0.59	−0.65	−1.22	−1.12	−1.26	1.22	1.02	0.22	0.69	−0.02	−0.26	−1.57
W3 vs. W2	1.47	0.80	0.45	0.57	1.35	−0.60	−0.65	−1.22	−1.12	−1.25	1.19	1.00	0.27	0.76	0.03	−0.26	−1.57
W3 vs. W3	1.52	0.79	0.38	0.59	1.30	−0.60	−0.66	−1.23	−1.13	−1.26	1.21	1.01	0.21	0.68	−0.03	−0.27	−1.57

The color gradient indicates the centrality values with blue colors indicating a lower degree of centrality and red ones indicating higher a degree of centrality. k, the rps11 gene; u, the rps21 gene.

a The mutated residues. For each cross type, the first lineage is given by the female parent and the second one by the paternal parent.

Looking at the result of the CCA measure, similar changes in centrality were observed for *rps11* compared with BCA results for both residues 116 and 117 (**Table 3**). Regarding CCA measures for *rps21*, similarly to BCA, loss of centrality was observed for residue 113 in this case, for the same crosses as BCA (**Table 3**).

We also observed changes in centrality (the DCA measure) for residue 98 (serine) of *rps11* in a unique way compared to the other measures: loss of centrality was observed for all crosses involving E1 as the mother plus crosses W3_W1 and W3_W2 (**Supplementary Table S3**). For the ECA centrality measure, for both *rps11* or *rps21* residues, no changes in centrality associated with cross type/direction were observed (**Supplementary Table S3**).

### 3.3 Principal component analysis

In order to investigate whether changes in centrality in genes *rps11–rps21* correlate with plastid–nuclear combinations, we looked at the distribution of the centrality of every residue of these gene pairs for the 16 models with PCA. In other words, considering the residue centralities as a multi-dimensional vector, we rotated this multi-dimensional space such that the primary axes aligned with the largest deviation. This allowed us to quantify how these vectors correlate for the 16 gene pair models (4×4). We only show the results of the PCA with BCA and CCA measures of centrality, these results being similar with the other three (cf. **Supplementary Data**).

**Figure 2:**
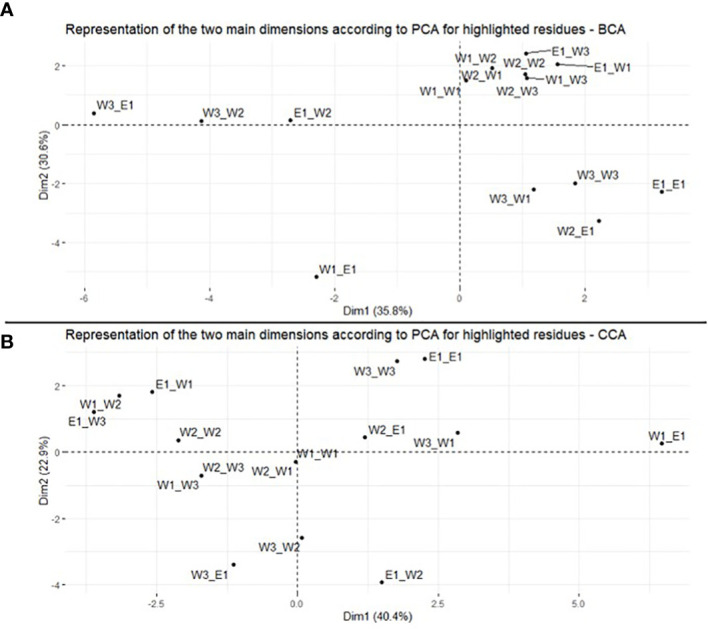
Principal component analysis of BCA **(A)** and CCA **(B)** of residues in the *rps11–rps21* genes. Representation of the 16 plastid–nuclear combinations on the two main dimensions.

Regarding the result for BCA, the first two dimensions of the PCA explained between 36% (Dim1) and 31% (Dim2) of the variance (i.e., 67% in total) (**Figure 2A**). Therefore, the centralities associated with the different cross types are discriminated by these two dimensions. Reciprocal crosses are well discriminated on the two first principal component (PC) axes (**Figure 2A**). Looking at centrality measures of cross E1_W2 and W2_E1, for example, they are found at different positions along PC1 and PC2. Similar results are observed, for example, between crosses E1_W1 (top right) and W1_E1 (bottom left) or between crosses W3_E1 (top left) and E1_W3 (top right), although PC2 does not discriminate the reciprocal crosses in this latter case (**Figure 2A**). For CCA results, PC1 and PC2 explained 40% and 23%, respectively (i.e., 63% in total) (**Figure 2B**). Similarly, in BCA, along the two main dimensions, reciprocal crosses clustered at different positions. For example, W3_W1 is located on the top right of the graph, while W1_W3 is located on the left side, in the middle (**Figure 2B**). A similar pattern is observed for W1_E1 (close to PC1, on the right) and E1_W1 (close to PC1, but on the left) or between W2_E1 (close to PC1 and PC2) and E1_W2 (on the other side of PC1) (**Figure 2B**). Similar results were observed with the other four measures of centrality (**Supplementary Figures S1, S2**).

**Figure 3:**
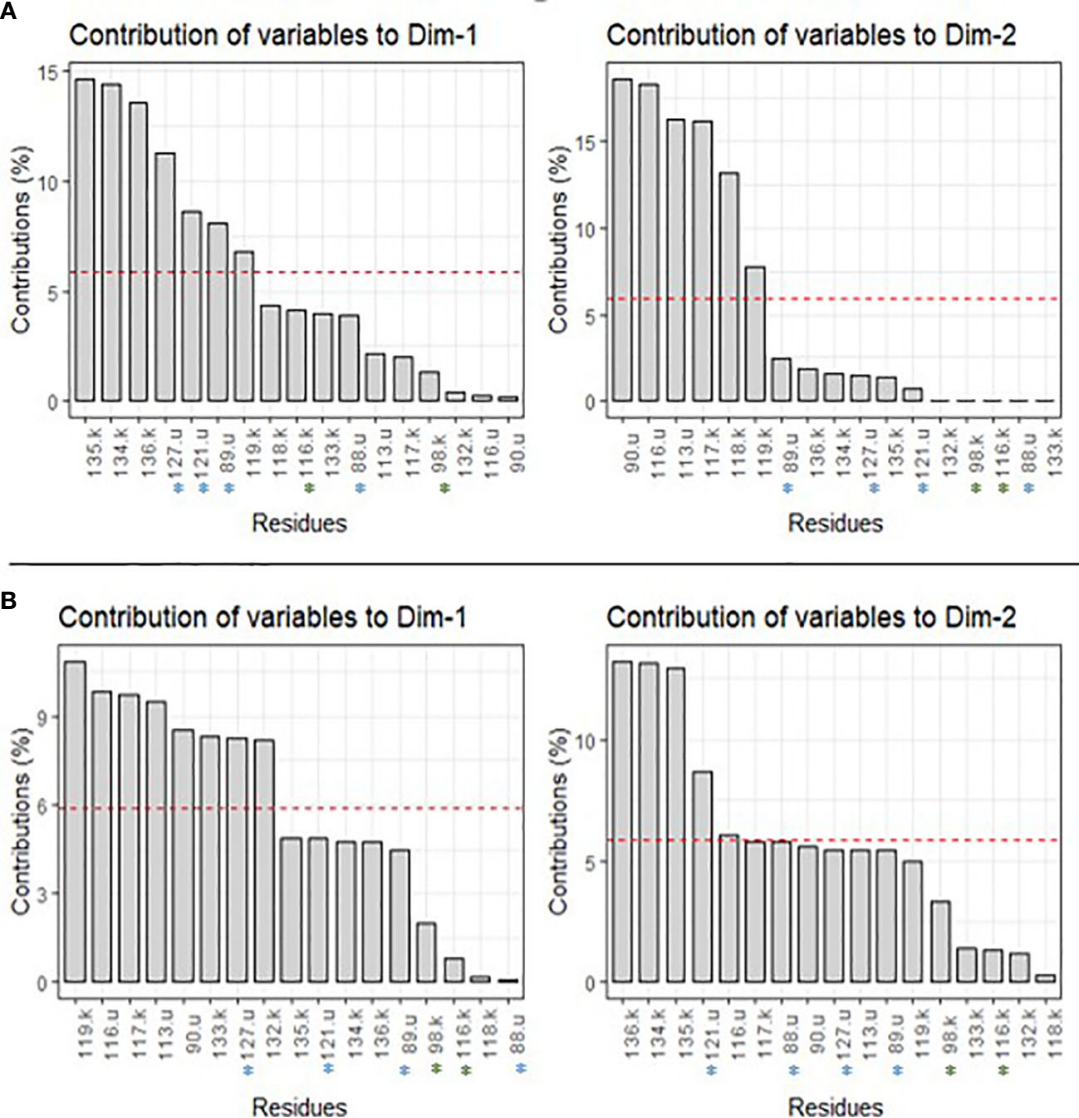
Contribution of each residue to PC1 (Dim1) and PC2 (Dim2) for BCA **(A)** and CCA **(B)** centrality measures. The red dashed line corresponds to the expected value if the contributions were uniform. u, residues of genes *rps21*; k, residues of gene *rps11*.

We also looked at the different contributions of each residue of *rps11* (*k*) and *rps21* (*u*) to the two main axes of variation on BCA and CCA (**Figure 3**; **Supplementary Table S4**). For BCA, for example, residues 134.*k*, 135.*k*, and 127.*u* contribute most for PC1 (i.e., 14.4%, 14.6%, and 11.3%, respectively) (**Figure 3**; **Supplementary Table S4**). For PC2, residues 117.*k*, 90.*u*, and 116.*u* contribute most (i.e., 16.1%, 18.6%, and 18.3%, respectively) (**Figure 3**; **Supplementary Table S4**). Regarding the CCA result, residues 119.*k*, 113.*u*, and 116.*u* contribute most to PC1 (i.e., 10.9%, 9.5%, and 9.8%, respectively), and residues 134.*k*, 136.*k*, and 121.*u* contribute most to PC2 (i.e., 13.1%, 13.2%, and 8.9%, respectively) (**Figure 3**; **Supplementary Table S4**). For the rest of the centrality measures, values are shown in **Supplementary Table S3**, but basically, some of the same residues contributed most to PC1 and PC2 (e.g., 135.*k* and 127.*u* for PC1 of DCA and ECA) (**Supplementary Table S4**).

## 4 Discussion

In the present study, we simulated mutations identified in different lineages of *S. nutans* and different genes coding for plastid ribosomal proteins on the three-dimensional structure of the spinach plastid ribosome to assess whether these mutations could (1) modify the interactions between ribosomal plastid and nuPt gene pairs and (2) impact the centrality of some of the interacting residues, further potentially impacting the structure of the ribosome. Some of the mutations identified did modify interactions between plastid and nuPt genes, either through the loss of existing interactions or the gaining of new ones. Focusing then on one specific plastid–nuclear gene pair (*rps11–rps21*), we assessed that these mutations, regardless of whether they modified the interactions, also impacted the centrality of some of these residues, differentially regarding the direction and type of cross between lineages (i.e., the 16 different plastid–nuclear combinations).

### 4.1 Impact of a nonfunctional plastid ribosome on plant fitness

Plastid ribosomes are an essential component for plant development and growth (Robles and Quesada, 2022), and plastid ribosome mutants might exhibit highly impaired phenotypes. For example, a study conducted on *Brassica campestris* ssp. *pekinensis* identified a missense mutation in a ribosomal plastid gene (*rps4*) causing aberrant rRNA processing, affecting plastid translation, and resulting in chlorophyll deficiency and reduced plant growth (Tang et al., 2018). Moreover, plastid ribosome is composed of proteins encoded by both nuclear (later called nuPt) and plastid genes, as is the case for all plastid complexes (Greiner and Bock, 2013). Some of these plastid and nuPt-encoded proteins interact together within the plastid ribosome at the residue level (Greiner and Bock, 2013; Forsythe et al., 2021). Because of differences in various evolutionary processes between these genomic compartments, tight co-evolution is required between interacting plastid and nuPt genes for proper plastid ribosome function (Greiner and Bock, 2013; Sloan et al., 2018), adding additional pressure on the appearance and consequences of any mutations occurring in plastid or nuPt genes encoding the plastid ribosome.

In *Silene nutans* lineages, we previously identified accumulation of substitutions in plastid and nuPt ribosomal genes mostly in lineages E1 and W2 (also the one displaying the highest seedling mortality rate in interlineage crosses) (Postel et al., 2022). This accumulation concerned essential plastid ribosomal genes: *rpl20*, *rpl22*, *rpl32*, *rps2*, *rps3*, *rps11*, and *rps18* (plastid genes) and *rpl3*, *rpl13*, *rpl21*, *rpl27*, *rps5*, *rps9*, and *rps13* (nuclear genes), which encode essential proteins for the function of the plastid ribosome (Tiller et al., 2012; Tiller and Bock, 2014). Knockout or dysfunction of one of these genes can lead to impaired, potentially nonviable mutant phenotypes (Tiller and Bock, 2014). The mutations identified in these essential ribosomal plastid genes could have important functional consequences. In the present study, some lineage-specific mutations in plastid or nuPt genes encoding the plastid ribosomal proteins lead to a change of the interactions between residue proteins within the large and small ribosomal subunit. If key interactions are disrupted, this could have subsequent consequences on important metabolic processes relying on the translational apparatus of the plant, e.g., the translation of photosynthetic proteins, essential to plastid function and plant development (Zoschke and Bock, 2018). Disruption of interaction between ribosomal proteins has already been shown to affect plant phenotypes (several listed in Robles and Quesada (2022)). For example, mutants for the ribosomal gene *rpl12* in rice species resulted in albino phenotypes and lethality at the seedling stage associated with low chlorophyll levels and misshapen plastid morphology, likely because the mutation in RPL12 abolished the interaction with another ribosomal protein, RPL10 (Zhao et al., 2016). Mutations identified here and leading to a modification of the interaction between residues of plastid and nuclear proteins might then be responsible for interlineage hybrid breakdown through disruption of co-adaptation between these plastid–nuclear gene pairs within the plastid ribosome, potentially destabilizing the function of the plastid ribosome and subsequently impacting interlineage hybrid development and growth (Robles and Quesada, 2022).

Focusing then on one plastid–nuclear gene pair, *rps11–rps21*, we identified cross-specific modification of the centrality of the residue of these genes, especially when lineage E1 was involved either as the mother or the father. *Rps11* is an essential plastid gene, as mentioned above, while *rps21* is not reported as such (Tiller and Bock, 2014). Yet, a recent study conducted on *rps21 Arabidopsis thaliana* mutants showed that a loss-of-function mutation in this nuPt genes resulted in defect in plant growth through photosynthesis defect and disruption of physiological response to carbon/nitrogen imbalance, highlighting its importance in plastid function (Dong et al., 2020). Modification of residue centralities in these two genes in crosses with lineage E1 might contribute to a modification of the protein network interaction. We also observed differences in centrality when lineage W3 is the mother, especially for residues of the plastid gene *rps11.* Though W3 does not lead to high percentage of hybrid mortality when used in interlineage crosses, it could nevertheless impact the whole small subunit ribosomal structure and its function. Overall, the different centrality calculations showed a loss or a gain of centrality according to the different mutations and crosses. The PCA results suggest that the centrality of the residues on the two main PC axes seems to reflect differences in hybrid breakdown between reciprocal crosses, as we observed in terms of interlineage hybrid fitness (Van Rossum et al., in prep). Especially regarding the contribution of the different residues to each PC for BCA and CCA, the results show that the centrality associated with interacting residues might be linked to the observed pattern of interlineage hybrid fitness of *Silene nutans*. For example, residues 135.*k* and 127.*u* contributed most to PC1–BCA. These residues are not mutated in the *rps11* sequence, but residue 127 of *rps21* is mutated for all western lineages compared to E1 (**Table 2**). Moreover, residues 127.*u* and 135.*k* are supposed to be in interaction within the small ribosomal subunit (**Table 2**). Even though the mutation in 127.*u* does not lead to a modification of the interaction between plastid and nuclear residues, it does seem to impact the centrality of these residues differentially when looking at reciprocal crosses. Similar results were observed between residues 136.*k* and 121.*u* and between 134.*k* and 121.*u* on PC2–CCA, with 121.*u* being mutated for lineage W2. The mutation of residue 121.*u* leads to the creation of a polar interaction with residue 134.*k* and no change of interaction with 136.*k*. In addition to the new interaction created, as previously, the mutation modified the centrality of these two residues, depending on the cross type and direction. This highlights the fact that these mutations could alter the centrality of protein residues and, through this, the residue network interactions between ribosomal proteins, with or without modifying the interactions, depending on the cross type and direction.

As shown in these studies, mutations in these essential ribosomal genes could impact the whole structure and function of the plastid ribosome through modification of interactions and centrality residue and the subsequent generation of PNIs in interlineage hybrids. As such, mutations in the plastid ribosome can be viewed as post-zygotic reproductive barriers. This could be further tested by assessing plastid translation in the interlineage hybrids or in plastome and nuclear mutants in tobacco where mutations found in *S. nutans* lineages could be inserted, mimicking interlineage hybrids (see (Malinova et al., 2021)).

### 4.2 Fast-evolving plastid genes involved in the plastid gene expression machinery and speciation

Plastid genes are supposed to be strongly conserved between species and evolve slowly because of strong selective constraints (Jansen et al., 2007). Yet, repeated observation of fast-evolving plastid genome have been described in several plant genera (*Silene* (Sloan et al., 2012; Sloan et al., 2014), *C. americanum* (Barnard-Kubow et al., 2014), *Geraniaceae* species (Ruhlman and Jansen, 2018)). Among them, this acceleration mostly concerned a subset of plastid genes: the one involved in the plastid gene expression machinery (i.e., the genes encoding the RNA polymerase complex and the plastid ribosome) and *accD* and *clpP1* (two other plastid genes involved in essential plastid function) (Weng et al., 2016; Williams et al., 2019; Forsythe et al., 2021).

Theoretically, these fast-evolving plastid genes would accumulate a larger amount of mutations than the slow-evolving ones. Increased accumulation of mutations would accelerate the plastid–nuclear co-evolution within these complexes (i.e., ACCase, CLP, RNA polymerase, and plastid ribosome). Lineages exhibiting such fast-evolving plastid genes would then potentially accumulate a larger amount of PNIs than other plant species with a more standard background rate of plastid evolution, under the condition of divergence without gene flow. Indeed, accumulation of mutations in these subsets of genes would theoretically increase the probability of having mutations modify the residue interactions or residue centrality within plastid complexes, as observed in our case. These modifications would then increase the probability of unstable, nonfunctional plastid–nuclear complexes in interlineage crosses. In our system, plastid genes have a usually high amount of mutations and relaxed selective pressure, suggesting that these genes might be fast-evolving (Postel et al., 2022). Moreover, these lineages experienced phases of divergence without gene flow during their isolation in independent glacial refugia (Martin et al., 2016; Van Rossum et al., 2018). Finally, in this study, we identified (1) mutations modifying essential interactions between plastid and nuPt genes encoding the plastid ribosome or (2) mutations modifying residue centrality, depending on the direction and type of the interlineage cross. Because it is essential to have a functional plastid ribosome for plant cell growth and development, the fast-evolving genes encoding the plastid ribosome of *Silene nutans* could be candidates for PNIs and could represent a strong post-zygotic reproductive barrier.

## 5 Conclusion

Only focusing on the most mutated gene pair, *rps11–rps21*, we showed that modification of residue centrality because of lineage-specific mutations seemed to be associated with cross type and direction.

To go further, this kind of study should be extended to the other mutations observed in plastid and nuPt genes encoding plastid ribosomal proteins (listed in **Supplementary Table S2**) that might also modify and disrupt the ribosome structure. Indeed, we cannot neglect that (1) the strength of the functional impact of a mutation and its associated structure modification may not follow a linear tendency: a few mutations impacting essential or central residues might also have strong functional consequences, and (2) we did not look at all at the interactions of the mutated plastid and nuclear proteins with the RNA, which could also have a functional impact.

The results of this study showed that some mutations impacted the interactions between some plastid and nuclear genes encoding the plastid ribosome, potentially modifying the whole structure of the plastid ribosome and its function in interlineage hybrids. It highlights that (1) modification of plastid–nuclear protein interactions or residue–contact centrality might influence the function of the plastid ribosome and that (2) because of its fast-evolving characteristic and the possibility of divergence without gene flow for these lineages, the plastid ribosome might be acting as post-zygotic reproductive barrier. This kind of approach appeared to be very useful to further identify gene pairs potentially inducing incompatibilities and their involvement as post-zygotic reproductive barriers. Combining genomic data analysis to identify mutations with modeling of these mutations on crystallographic structure represents a good methodology to identify which of the mutations effectively modify interactions between interacting genes and which of these interactions’ modifications can have an impact on the whole complex structure and, by doing so, its function.

## Data availability statement

Publicly available datasets were analyzed in this study. This data can be found here: National center for Biotechnology Information (NCBI) BioProject, https://www.ncbi.nlm.nih.gov/bioproject/ , PRJNA745523.

## Author contributions

ZP: investigation, visualization, and writing—original draft.TM: investigation, formal analysis, visualization, and writing—original draft.ML: methodology, supervision, and writing—review and editing. PT: conceptualization, funding acquisition, supervision, and writing—review and editing. All authors contributed to the article and approved the submitted version.
