# Supplementary material for: What is the potential impact of genetic divergence of plastid ribosomal genes between Silene nutans lineages in hybrids? An in silico approach using the 3D structure of the plastid ribosome
Source: Front Plant Sci. 2023 May 8;14:1167478. doi: 10.3389/fpls.2023.1167478 (PMC10201985; doi:10.3389/fpls.2023.1167478)
Supplement: Supplementary file 1 [file DataSheet_1.docx]

| **Suppl. Table S1**  List of the nuclear and plastid genes encoding the large ribosomal subunit, selected as candidates for PNIs in Postel et al, 2022 and analyzed in the present study. | | | | | | | | | | | |
| --- | --- | --- | --- | --- | --- | --- | --- | --- | --- | --- | --- |
| **Genome** | **Gene name** | **UniProt identifier** | **Chain's name** | **Entitled** | ***Spinacia oleracea*** | | ***S. nutans* lineages** | | | | |
|  |  |  |  |  | **Position** | **Amino Acid** | **Position** | **Amino acid** | | | |
|  |  |  |  |  |  |  |  | **E1** | **W1** | **W2** | **W3** |
| Nuclear | *rpl13* | P12629 | K | S | 132 | **A** | 152 | **A** | **S** | **A** | **A** |
|  | *rpl19* | P82413 | Q | Y | 229 | **L** | 235 | **F** | **L** | **L** | **L** |
|  | *rpl21* | P24613 | S | AA | 32 | **P** | 61 | **R** | **K** | **K** | **K** |
|  | *rpl27* | P82190 | X | FA | 20 | **L** | 20 | **L** | **L** | **V** | **L** |
|  | *rpl3* | P82191 | D | L | 35 | **S** | 32 | **S** | **F** | **F** | **F** |
| Plastid | *rpl14* | P09596 | L | T | 49 | **N** | 49 | **N** | **H** | **H** | **H** |
|  |  |  |  |  | 104 | **R** | 104 | **G** | **R** | **R** | **R** |
|  | *rpl16* | P17353 | N | V | 26 | **R** | 24 | **N** | **N** | **T** | **N** |
|  |  |  |  |  | 78 | **P** | 76 | **P** | **P** | **S** | **P** |
|  | *rpl22* | P09594 | T | BA | 9 | **K** | 6 | **R** | **G** | **R** | **R** |
|  |  |  |  |  | 114 | **V** | 92 | **L** | **F** | **L** | **L** |
|  |  |  |  |  | 115 | **K** | 93 | **K** | **N** | **N** | **N** |
|  |  |  |  |  | 121 | **R** | 99 | **R** | **H** | **H** | **H** |
|  | *rpl32* | P28804 | 1 | B | 22 | **K** | 22 | **K** | **K** | **M** | **K** |
|  |  |  |  |  | 28 | **A** | 28 | **A** | **A** | **V** | **A** |
|  |  |  |  |  | 49 | **R** | 49 | **L** | **L** | **P** | **L** |
| In green: different amino-acid between *S. oleracea* and *S. nutans* lineages ; in purple: different amino-acid for one or more lineages of *S. nutans* compared to *S. oleracea* ; in orange: different amino-acid for one lineage of *S. nutans* compared to the others and to *S. oleracea.* | | | | | | | | | | | |

| **Suppl. Table S2**  List of the nuclear and plastid genes encoding the small ribosomal subunit, selected as candidates for PNIs in Postel et al, 2022 and analyzed in the present study. | | | | | | | | | | | | | |
| --- | --- | --- | --- | --- | --- | --- | --- | --- | --- | --- | --- | --- | --- |
| **Genome** | **Gene name** | **UniProt identifier** | | **Chain's name** | **Entitled** | | ***Spinacia oleracea*** | | ***S. nutans* lineages** | | | | |
|  |  |  |  |  |  |  | **Position** | **Amino Acid** | **Position** | **Amino acid** | | | |
|  |  |  |  |  |  |  |  |  |  | **E1** | **W1** | **W2** | **W3** |
| Nuclear | *rps10* | | P82162 | j | | TA | 77 | **D** | 82* | **E** | **E** | **E** | **K** |
|  |  |  |  |  |  |  | 142 | **Y** | 147 | **F** | **F** | **F** | **Y** |
|  | *rps13* | | P82163 | m | | WA | 127 | **E** | 125* | **E** | **E** | **E** | **Q** |
|  | *rps21* | | P82024 | u | | EB | 88 | **V** | 36 | **I** | **V** | **V** | **V** |
|  |  |  |  |  |  |  | 89 | **L** | 37 | **F** | **L** | **L** | **S** |
|  |  |  |  |  |  |  | 91 | **Q** | 39 | **N** | **D** | **D** | **D** |
|  |  |  |  |  |  |  | 133 | **H** | 81 | **S** | **A** | **S** | **S** |
|  |  |  |  |  |  |  | 157 | **E** | 106 | **K** | **E** | **E** | **E** |
|  |  |  |  |  |  |  | 121 | **Y** | 51 | **H** | **H** | **Y** | **H** |
|  |  |  |  |  |  |  | 127 | **E** | 57 | **E** | **D** | **D** | **D** |
|  |  |  |  |  |  |  | 156 | **E** | 85 | **D** | **D** | **A** | **D** |
|  |  |  |  |  |  |  | 62 | **G** | 69 | **S** | **T** | **T** | **S** |
|  |  |  |  |  |  |  | 135 | **E** | 134 | **E** | **D** | **D** | **D** |
|  | *rps5* | | Q9ST69 | e | | OA | 77 | **K** | 67 | **R** | **R** | **R** | **Q** |
|  |  |  |  |  |  |  | 141 | **S** | 131 | **T** | **T** | **T** | **S** |
|  |  |  |  |  |  |  | 172 | **M** | 183* | **L** | **L** | **M** | **L** |
|  |  |  |  |  |  |  | 198 | **V** | 209 | **V** | **V** | **V** | **I** |
|  | *rps6* | | P82403 | f | | PA | 62 | **A** | 48 | **A** | **T** | **T** | **T** |
|  |  |  |  |  |  |  | 149 | **V** | 134 | **L** | **V** | **V** | **V** |
|  |  |  |  |  |  |  | 157 | **K** | 142 | **N** | **N** | **N** | **I** |
|  |  |  |  |  |  |  | 162 | **A** | 147 | **A** | **E** | **E** | **E** |
| Plastid | *rps11* | | P06506 | k | | UA | 6 | **P** | 6 | **L** | **P** | **P** | **P** |
|  |  |  |  |  |  |  | 13 | **N** | 13 | **K** | **N** | **N** | **Y** |
|  |  |  |  |  |  |  | 76 | **A** | 76 | **T** | **A** | **A** | **A** |
|  |  |  |  |  |  |  | 78 | **N** | 78 | **D** | **D** | **N** | **D** |
|  |  |  |  |  |  |  | 82 | **T** | 82 | **T** | **T** | **P** | **T** |
|  |  |  |  |  |  |  | 98 | **P** | 95 | **P** | **S** | **S** | **P** |
|  |  |  |  |  |  |  | 98 | **P** | 96 | **P** | **S** | **S** | **P** |
|  |  |  |  |  |  |  | 98 | **P** | 98 | **P** | **S** | **S** | **P** |
|  |  |  |  |  |  |  | 104 | **A** | 104 | **A** | **A** | **A** | **G** |
|  |  |  |  |  |  |  | 108 | **A** | 108 | **V** | **A** | **A** | **A** |
|  |  |  |  |  |  |  | 114 | **I** | 114 | **I** | **L** | **L** | **L** |
|  |  |  |  |  |  |  | 116 | **L** | 116 | **L** | **L** | **L** | **V** |
|  | *rps18* | | Q9M3K7 | r | | BB | 13 | **R** | 14 | **R** | **R** | **R** | **Q** |
|  |  |  |  |  |  |  | 18 | **R** | 17 | **H** | **R** | **R** | **R** |
|  |  |  |  |  |  |  | 50 | **R** | 49 | **R** | **R** | **Q** | **R** |
|  |  |  |  |  |  |  | 81 | **E** | 80 | **-** | **R** | **G** | **G** |
|  |  |  |  |  |  |  | 81 | **E** | 82 | **-** | **R** | **G** | **G** |
|  |  |  |  |  |  |  | 94 | **A** | 93 | **-** | **I** | **Q** | **I** |
|  | *rps19* | | P06508 | s | | CB | 19 | **I** | 19 | **M** | **M** | **M** | **I** |
|  |  |  |  |  |  |  | 33 | **T** | 33 | **T** | **T** | **T** | **N** |
|  |  |  |  |  |  |  | 65 | **R** | 65* | **R** | **R** | **Y** | **D** |
|  |  |  |  |  |  |  | 91 | **R** | 91 | **R** | **R** | **R** | **Q** |
|  | *rps2* | | P08242 | b | | LA | 24 | **T** | 24 | **I** | **I** | **T** | **I** |
|  | *rps3* | | P09595 | c | | MA | 79 | **G** | 79 | **G** | **A** | **G** | **G** |
|  |  |  |  |  |  |  | 94 | **D** | 94 | **D** | **D** | **A** | **D** |
|  |  |  |  |  |  |  | 103 | **L** | 103 | **L** | **L** | **F** | **L** |
|  |  |  |  |  |  |  | 117 | **I** | 117 | **I** | **I** | **I** | **L** |
|  |  |  |  |  |  |  | 213 | **I** | 213 | **I** | **I** | **I** | **L** |
|  | *rps7* | | P82129 | g | | QA | 151 | **F** | 151* | **F** | **L** | **L** | **F** |
| In green: different amino-acid between *S. oleracea* and *S. nutans* lineages ; in purple: different amino-acid for one or more lineages of *S. nutans* compared to *S. oleracea* ; in orange: different amino-acid for one lineage of *S. nutans* compared to the others and to *S. oleracea ;* * : mutations identified as under positive selection in Postel et al, 2022 ; “-“: indicate absence of amino-acid in the multiple sequence alignment from Postel et al. 2022. | | | | | | | | | | | | | |

| **Suppl. Table S3** | | | | | | | | | | | | | | | | | |
| --- | --- | --- | --- | --- | --- | --- | --- | --- | --- | --- | --- | --- | --- | --- | --- | --- | --- |
| BCA and CCA centrality score of mutated and interacting residues of *rps11 and rps21* according to the 16 different plastid-nuclear combinations. The color gradient indicates the centrality values with blue colors indicating the lower degree of centrality and red ones’ higher degree of centrality. | | | | | | | | | | | | | | | | | |
| **DCA** | | | | | | | | | | | | | | | | | |
| **Crosses** | ***rps11*** | | | | | | | | | | ***rps21*** | | | | | | |
|  | **98.k*** | **116.k*** | **117.k** | **118.k** | **119.k** | **132.k** | **133.k** | **134.k** | **135.k** | **136.k** | **88.u*** | **89.u*** | **90.u** | **113.u** | **116.u** | **121.u*** | **127.u*** |
| E1 vs E1 | -0.21 | 1.15 | 0.13 | 0.47 | 1.49 | -0.55 | -0.89 | -0.89 | -0.55 | -1.56 | -0.21 | 0.47 | 0.47 | 0.47 | -0.89 | 0.47 | 0.13 |
| E1 vs W1 | -0.21 | 1.14 | 0.46 | 0.46 | 1.48 | -0.55 | -0.89 | -0.89 | -0.55 | -1.57 | -0.21 | 0.46 | 0.46 | 0.46 | -0.89 | 0.46 | 0.13 |
| E1 vs W2 | -0.20 | 1.16 | 0.48 | 0.48 | 1.50 | -0.54 | -0.88 | -0.88 | -0.54 | -1.56 | -0.20 | 0.48 | 0.48 | 0.48 | -0.88 | 0.48 | 0.14 |
| E1 vs W3 | -0.22 | 1.14 | 0.46 | 0.46 | 1.49 | -0.56 | -0.90 | -0.90 | -0.56 | -1.59 | -0.22 | 0.46 | 0.46 | 0.46 | -0.90 | 0.46 | 0.12 |
| W1 vs E1 | 0.13 | 1.15 | 0.13 | 0.47 | 1.49 | -0.55 | -0.89 | -0.89 | -0.55 | -1.57 | -0.21 | 0.47 | 0.47 | 0.47 | -0.89 | 0.47 | 0.13 |
| W1 vs W1 | 0.12 | 1.13 | 0.46 | 0.46 | 1.47 | -0.55 | -0.89 | -0.89 | -0.55 | -1.56 | -0.21 | 0.46 | 0.46 | 0.46 | -0.89 | 0.46 | 0.12 |
| W1 vs W2 | 0.12 | 1.15 | 0.46 | 0.46 | 1.49 | -0.56 | -0.90 | -0.90 | -0.56 | -1.58 | -0.22 | 0.46 | 0.46 | 0.46 | -0.90 | 0.46 | 0.12 |
| W1 vs W3 | 0.12 | 1.14 | 0.46 | 0.46 | 1.47 | -0.55 | -0.89 | -0.89 | -0.55 | -1.57 | -0.22 | 0.46 | 0.46 | 0.46 | -0.89 | 0.46 | 0.12 |
| W2 vs E1 | 0.12 | 1.14 | 0.12 | 0.46 | 1.48 | -0.57 | -0.91 | -0.91 | -0.57 | -1.59 | -0.23 | 0.46 | 0.46 | 0.46 | -0.91 | 0.46 | 0.12 |
| W2 vs W1 | 0.12 | 1.13 | 0.46 | 0.46 | 1.47 | -0.55 | -0.89 | -0.89 | -0.55 | -1.56 | -0.21 | 0.46 | 0.46 | 0.46 | -0.89 | 0.46 | 0.12 |
| W2 vs W2 | 0.12 | 1.13 | 0.46 | 0.46 | 1.47 | -0.56 | -0.90 | -0.90 | -0.56 | -1.57 | -0.22 | 0.46 | 0.46 | 0.46 | -0.90 | 0.46 | 0.12 |
| W2 vs W3 | 0.12 | 1.14 | 0.46 | 0.46 | 1.47 | -0.55 | -0.89 | -0.89 | -0.55 | -1.57 | -0.22 | 0.46 | 0.46 | 0.46 | -0.89 | 0.46 | 0.12 |
| W3 vs E1 | 0.14 | 0.82 | 0.48 | 0.48 | 1.51 | -0.55 | -0.89 | -0.89 | -0.55 | -1.58 | -0.21 | 0.48 | 0.48 | 0.48 | -0.89 | 0.48 | 0.14 |
| W3 vs W1 | -0.20 | 1.16 | 0.14 | 0.48 | 1.50 | -0.54 | -0.88 | -0.88 | -0.54 | -1.56 | -0.20 | 0.48 | 0.48 | 0.48 | -0.88 | 0.48 | 0.14 |
| W3 vs W2 | -0.21 | 1.15 | 0.47 | 0.47 | 1.49 | -0.55 | -0.88 | -0.88 | -0.55 | -1.56 | -0.21 | 0.47 | 0.47 | 0.47 | -0.88 | 0.47 | 0.13 |
| W3 vs W3 | 0.13 | 0.81 | 0.13 | 0.47 | 1.49 | -0.55 | -0.89 | -0.89 | -0.55 | -1.57 | -0.21 | 0.47 | 0.47 | 0.47 | -0.89 | 0.47 | 0.13 |
| **ECA** | | | | | | | | | | | | | | | | | |
| **Crosses** | ***rps11*** | | | | | | | | | | ***rps21*** | | | | | | |
|  | **98.k*** | **116.k*** | **117.k** | **118.k** | **119.k** | **132.k** | **133.k** | **134.k** | **135.k** | **136.k** | **88.u*** | **89.u*** | **90.u** | **113.u** | **116.u** | **121.u*** | **127.u*** |
| E1 vs E1 | 0.10 | 1.39 | 0.35 | 0.63 | 1.75 | -1.20 | -1.22 | -1.25 | -1.25 | -1.27 | -0.17 | -0.44 | 0.50 | 0.83 | -0.27 | -1.14 | -1.26 |
| E1 vs W1 | 0.09 | 1.43 | 0.50 | 0.67 | 1.78 | -1.21 | -1.23 | -1.26 | -1.26 | -1.28 | -0.18 | -0.45 | 0.52 | 0.85 | -0.25 | -1.14 | -1.27 |
| E1 vs W2 | 0.11 | 1.42 | 0.48 | 0.65 | 1.76 | -1.20 | -1.22 | -1.25 | -1.25 | -1.27 | -0.18 | -0.44 | 0.51 | 0.81 | -0.26 | -1.13 | -1.26 |
| E1 vs W3 | 0.09 | 1.53 | 0.53 | 0.75 | 1.89 | -1.19 | -1.21 | -1.24 | -1.24 | -1.26 | -0.17 | -0.44 | 0.58 | 0.89 | -0.21 | -1.13 | -1.25 |
| W1 vs E1 | 0.28 | 1.39 | 0.36 | 0.64 | 1.77 | -1.20 | -1.23 | -1.26 | -1.26 | -1.28 | -0.14 | -0.41 | 0.50 | 0.84 | -0.27 | -1.14 | -1.27 |
| W1 vs W1 | 0.26 | 1.43 | 0.48 | 0.68 | 1.80 | -1.19 | -1.21 | -1.24 | -1.24 | -1.26 | -0.16 | -0.43 | 0.51 | 0.83 | -0.26 | -1.13 | -1.25 |
| W1 vs W2 | 0.24 | 1.51 | 0.52 | 0.73 | 1.86 | -1.19 | -1.21 | -1.24 | -1.24 | -1.25 | -0.18 | -0.45 | 0.57 | 0.86 | -0.22 | -1.13 | -1.25 |
| W1 vs W3 | 0.26 | 1.45 | 0.47 | 0.68 | 1.78 | -1.19 | -1.21 | -1.24 | -1.24 | -1.26 | -0.18 | -0.44 | 0.49 | 0.78 | -0.27 | -1.13 | -1.25 |
| W2 vs E1 | 0.32 | 1.43 | 0.35 | 0.65 | 1.71 | -1.21 | -1.23 | -1.26 | -1.26 | -1.28 | -0.16 | -0.44 | 0.49 | 0.74 | -0.28 | -1.14 | -1.27 |
| W2 vs W1 | 0.26 | 1.43 | 0.48 | 0.68 | 1.80 | -1.19 | -1.21 | -1.24 | -1.24 | -1.26 | -0.16 | -0.43 | 0.51 | 0.83 | -0.26 | -1.13 | -1.25 |
| W2 vs W2 | 0.25 | 1.46 | 0.48 | 0.69 | 1.81 | -1.19 | -1.21 | -1.24 | -1.24 | -1.26 | -0.18 | -0.44 | 0.53 | 0.82 | -0.25 | -1.13 | -1.25 |
| W2 vs W3 | 0.26 | 1.45 | 0.47 | 0.68 | 1.78 | -1.19 | -1.21 | -1.24 | -1.24 | -1.26 | -0.18 | -0.44 | 0.49 | 0.78 | -0.27 | -1.13 | -1.25 |
| W3 vs E1 | 0.27 | 1.11 | 0.47 | 0.69 | 1.82 | -1.19 | -1.22 | -1.25 | -1.25 | -1.26 | -0.15 | -0.41 | 0.52 | 0.87 | -0.23 | -1.13 | -1.26 |
| W3 vs W1 | 0.12 | 1.28 | 0.39 | 0.71 | 1.82 | -1.18 | -1.21 | -1.23 | -1.23 | -1.25 | -0.16 | -0.42 | 0.53 | 0.86 | -0.23 | -1.12 | -1.25 |
| W3 vs W2 | 0.10 | 1.30 | 0.53 | 0.73 | 1.80 | -1.20 | -1.22 | -1.25 | -1.25 | -1.27 | -0.19 | -0.45 | 0.51 | 0.82 | -0.25 | -1.14 | -1.26 |
| W3 vs W3 | 0.32 | 1.03 | 0.30 | 0.62 | 1.75 | -1.19 | -1.22 | -1.24 | -1.24 | -1.26 | -0.13 | -0.38 | 0.46 | 0.81 | -0.27 | -1.12 | -1.26 |
| “k” : the *rps11* gene ; "u": the *rps21* gene ; *: the mutated residues ; for each cross type, the first lineage given in the female parent and the second one the paternal parent. | | | | | | | | | | | | | | | | | |

**Suppl. Fig. S1 Principal Component Analysis of DCA of residues in the *rps11-rps21* genes.** Representation of the 16 cross types and directions on the two main dimensions.


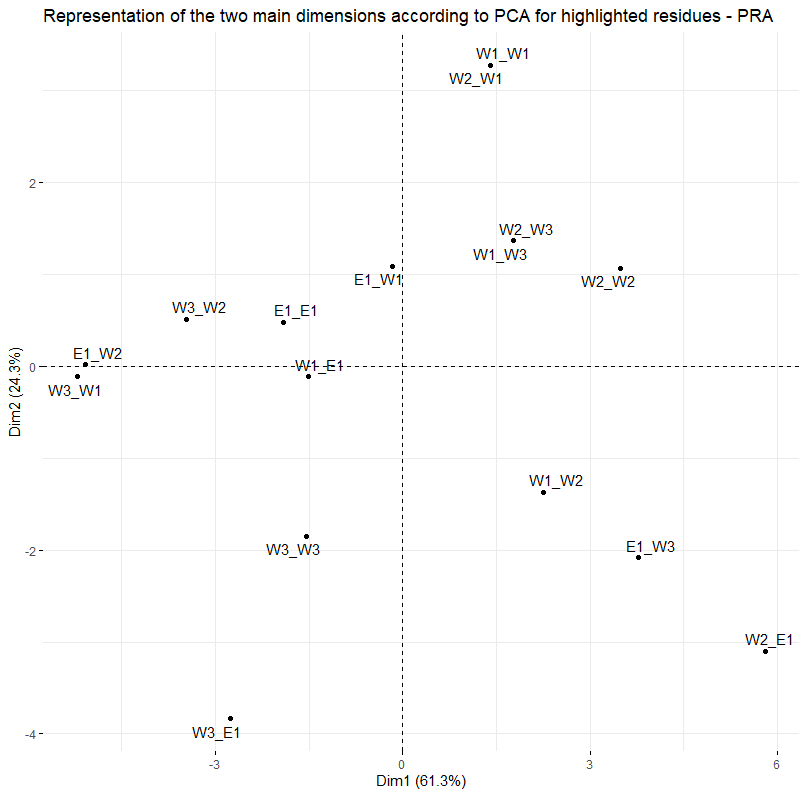


**Suppl. Fig. S2 Principal Component Analysis of ECA of residues in the *rps11-rps21* genes.** Representation of the 16 cross types and directions on the two main dimensions.


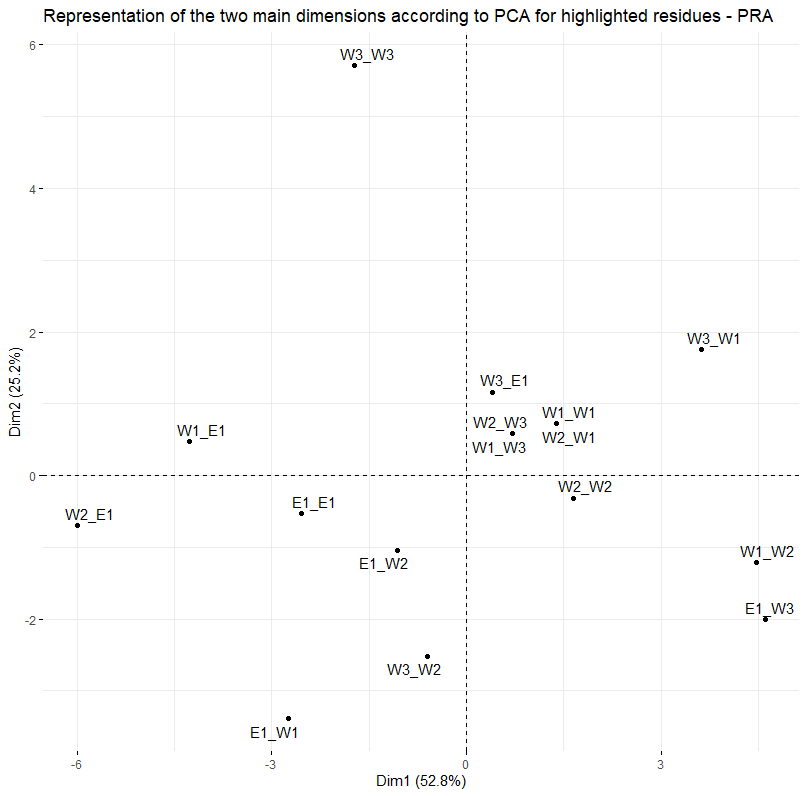


| **Suppl. Table S4**  Contribution of each residue to the three main principal components for each centrality measure. In green and blue are the plastid and nuclear residues, respectively, that contribute the most to each principal component. | | | | | | | | | | | | | |  |
| --- | --- | --- | --- | --- | --- | --- | --- | --- | --- | --- | --- | --- | --- | --- |
| **Gene** | **Residue** | **BCA** | | | **CCA** | | | **DCA** | | | **ECA** | | |  |
|  |  | **PC1** | **PC2** | **PC3** | **PC1** | **PC2** | **PC3** | **PC1** | **PC2** | **PC3** | **PC1** | **PC2** | **PC3** | |
| ***rps21*** | 88* | 3.895 | 0.017 | **24.712** | 0.069 | 5.800 | **24.076** | **9.482** | 0.265 | 0.070 | 0.781 | 15.016 | 11.197 | |
|  | 89* | 8.096 | 2.481 | 3.112 | 4.452 | 5.414 | 3.545 | 7.864 | 4.283 | 0.048 | 0.319 | **17.543** | 9.408 | |
|  | 90 | 0.161 | **18.596** | 0.013 | 8.540 | 5.555 | 2.235 | 7.864 | 4.283 | 0.048 | 4.890 | 7.533 | 6.258 | |
|  | 113 | 2.154 | 16.241 | 0.008 | **9.479** | 5.439 | 0.218 | 7.864 | 4.283 | 0.048 | 3.588 | 0.673 | **27.22** | |
|  | 116 | 0.283 | **18.253** | 0.502 | **9.830** | 6.035 | 0.078 | 5.625 | **9.833** | **0.535** | 6.657 | 1.964 | 13.437 | |
|  | 121* | 8.645 | 0.737 | 14.96 | 4.848 | **8.696** | 7.889 | 7.864 | 4.283 | 0.048 | 7.203 | 7.628 | 0.034 | |
|  | 127* | **11.266** | 1.512 | 0.001 | 8.286 | 5.451 | 3.724 | **9.231** | 0.890 | 0.000 | **9.544** | 1.855 | 2.606 | |
| ***rps11*** | 98* | 1.296 | 0.059 | **13.488** | 1.996 | 3.316 | 2.469 | 2.080 | 0.000 | **52.129** | 0.44 | 8.397 | 3.227 | |
|  | 116* | 4.107 | 0.059 | 10.965 | 0.814 | 1.322 | **23.085** | 0.383 | 6.152 | 44.012 | 0.463 | **10.681** | **10.02** | |
|  | 117 | 2.004 | **16.144** | 0.482 | 9.752 | 5.810 | 0.026 | 0.199 | 2.252 | 0.271 | 3.784 | 9.800 | 0.677 | |
|  | 118 | 4.350 | 13.198 | 0.945 | 0.177 | 0.329 | 2.046 | **7.864** | 4.283 | 0.048 | 6.486 | 4.885 | 0.42 | |
|  | 119 | 6.779 | 7.798 | 4.788 | **10.868** | 4.973 | 0.156 | 4.213 | 13.34 | 0.321 | 8.668 | 1.065 | 6.004 | |
|  | 132 | 0.434 | 0.074 | 12.448 | 8.228 | 1.205 | 7.882 | **7.984** | 3.984 | 0.28 | 8.881 | 4.073 | 1.057 | |
|  | 133 | 4.003 | 0.010 | 11.629 | 8.297 | 1.368 | 7.787 | 5.625 | 9.833 | 0.535 | **9.285** | 3.048 | 1.679 | |
|  | 134 | **14.373** | 1.563 | 0.445 | 4.764 | **13.139** | 4.876 | 5.625 | 9.833 | 0.535 | **9.626** | 2.115 | 2.099 | |
|  | 135 | **14.635** | 1.415 | 0.001 | 4.859 | 12.924 | 5.163 | **7.984** | 3.984 | 0.280 | **9.608** | 2.150 | 2.087 | |
|  | 136 | 13.518 | 1.843 | 1.501 | 4.741 | **13.222** | 4.743 | 2.249 | **18.216** | 0.790 | **9.776** | 1.575 | 2.569 | |
| *: the residues that are mutated in at least one lineage of *S. nutans*. | | | | | | | | | | | | | | |
